# Intra-articular injection of gold micro-particles with hyaluronic acid for painful knee osteoarthritis

**DOI:** 10.1186/s12891-024-07321-4

**Published:** 2024-03-12

**Authors:** Sten Rasmussen, Kristian Kjaer Petersen, Christopher Aboo, Jacob Skallerup Andersen, Emilie Skjoldemose, Nia Kristine Jørgensen, Allan Stensballe, Lars Arendt-Nielsen

**Affiliations:** 1 Department of Clinical Medicine, Aalborg University, 249 Selma Lagerløfs Vej, Gistrup, 9260 Denmark; 2 Department of Orthopedic Surgery, Sport and Arthroscopy, Aalborg University Hospital, Aalborg, Denmark; 3 Department of Health Science and Technology, Aalborg University, Aalborg, Denmark; 4 Center for Neuroplasticity and Pain, Department of Health Science and Technology, Faculty of Medicine, Aalborg University, Aalborg, Denmark; 5 Clinical Cancer Research Center, Aalborg University Hospital, Aalborg, Denmark; 6 Department of Gastroenterology & Hepatology, Mech-Sense, Aalborg University Hospital, Aalborg, Denmark; 7 Steno Diabetes Center North Denmark, Aalborg University Hospital, Aalborg, Denmark

**Keywords:** Gold microparticles, Hyaluronic acid, Intra-articular injection, Knee osteoarthritis, Outcomes

## Abstract

**Background:**

Recently, in an open pilot study, we found up to two years, a potential pain-relieving effect of intra-articular gold micro-particles using the patient’s synovial fluid for patients with knee osteoarthritis (KOA). During the study the excluded group of patients, due to multisite pain, co-morbidities, and other exclusion criteria., received intra-articular gold micro-particles using hyaluronic acid,. We aimed to identify if pre-treatment characteristics influence the global outcome two years after intra-articular treatment for painful KOA with gold microparticles using hyaluronic acid.

**Methods:**

Using hyaluronic acid as the carrier, 136 patients with KOA received intraarticular injections with 20 mg gold microparticles (72.000 particles, 20–40 μm in diameter). In the analysis, we included the Global Rating of Change Scale, Pain Detect Questionnaire (PDQ), Body Mass Index (BMI), and Kellgren & Lawrence score at the inclusion, Western Ontario, and McMaster Universities Osteoarthritis Index (WOMAC) sub-scores for pain, stiffness, and function at inclusion and two years.

**Results:**

On the Global Rating Change Scale, 69.1% of patients reported a positive effect, 28.7% no effect, and 2.2% worse. PDQ and the three WOMAC subscores all improved at two years of follow-up. PDQ ≥ 13 (*P* = 0.028), BMI (*P* = 0.022) and Kellgren & Lawrence grade 4 (*P* = 0.028) at inclusion reduced the effect with a minor odds ratio compared to the baseline effect of treatment (*P* = 0.025). WOMAC subscores at inclusion did not influence the outcome (*P* > 0.5).

**Conclusions:**

Severe osteoarthritis, obesity, and neuropathic pain, reduced the effect of intra-articular gold microparticles for knee OA.

**Trial registration:**

The study followed the principles of the Declaration of Helsinki and was approved by the local ethics committee of the North Denmark Region by 27/07/2016 (N-20,160,045). The regional data protection agency approved the project by 06/07/2016 (2008-58-0028, ID 2016 − 116) and registered in ClinicalTrial.Gov by 04/01/2018 (NCT03389906).

## Introduction

Intra-articular treatment with steroids, hyaluronic acid, and platelet-rich fibrin provides in some cases temporary relief from pain and improvement of function in knee osteoarthritis (KOA) [1–3]. In an open pilot study, we recently found a longer-acting, up to two years, pain-relieving effect of intra-articular gold micro-particles for patients with knee osteoarthritis (KOA). The observed pain-reducing effect appears to be partly a result of immunosuppression and regenerative processes [4].

Patients with individual risk factors, including the preoperative degree of osteoarthritis, pain, function smoking, deprivation, obesity, and the use of opioids, achieve poor outcomes after knee replacement arthroplasty [5–8]. Several studies indicate an association between signs of pain sensitization as a predictor of chronic postoperative pain [9–11] and chronic residual pain after total joint replacement [12–14]. A recent review reports central neurophysiological changes in patients with KOA and recommends that more neuroplasticity studies are needed to prove this association [15]. The Pain Detect Questionnaire (PDQ) might be a surrogate assessment for pain sensitization [16] and preoperative PDQ is associated with chronic postoperative pain after total knee arthroplasty [17]. A cut-off at 13 points on PDQ indicate a possible neuropathic pain component [18, 19] and may determine a poorer outcome after intra-articular treatment with gold micro-particles. It is important to identify the patients at risk of poor patient-reported outcome and to evaluate the risks and benefits of intra-articular gold micro-particle injections.

During the inclusion of our open pilot study, we excluded a larger group of KOA patients due to multisite pain, co-morbidities, and other exclusion criteria [4]. This group received intra-articular gold micro-particles using hyaluronic acid, whereas the included group received the treatment using the patient’s synovial fluid. The question for this study is whether pre-treatment characteristics influence the global outcome two years after intra-articular treatment for painful KOA with gold microparticles using hyaluronic acid.

This study aims to identify if pre-treatment characteristics influence the global outcome two years after intra-articular treatment of painful knee OA patients with gold micro-particles.

## Methods

### Study flow

We evaluated pain and function at baseline, after eight weeks, and at a two-year follow in two groups of patients with painful KOA. The patients received intra-articular 20 mg gold micro-particles using hyaluronic acid (HA) as the carrier.

### Participants

**Fig. 1:**
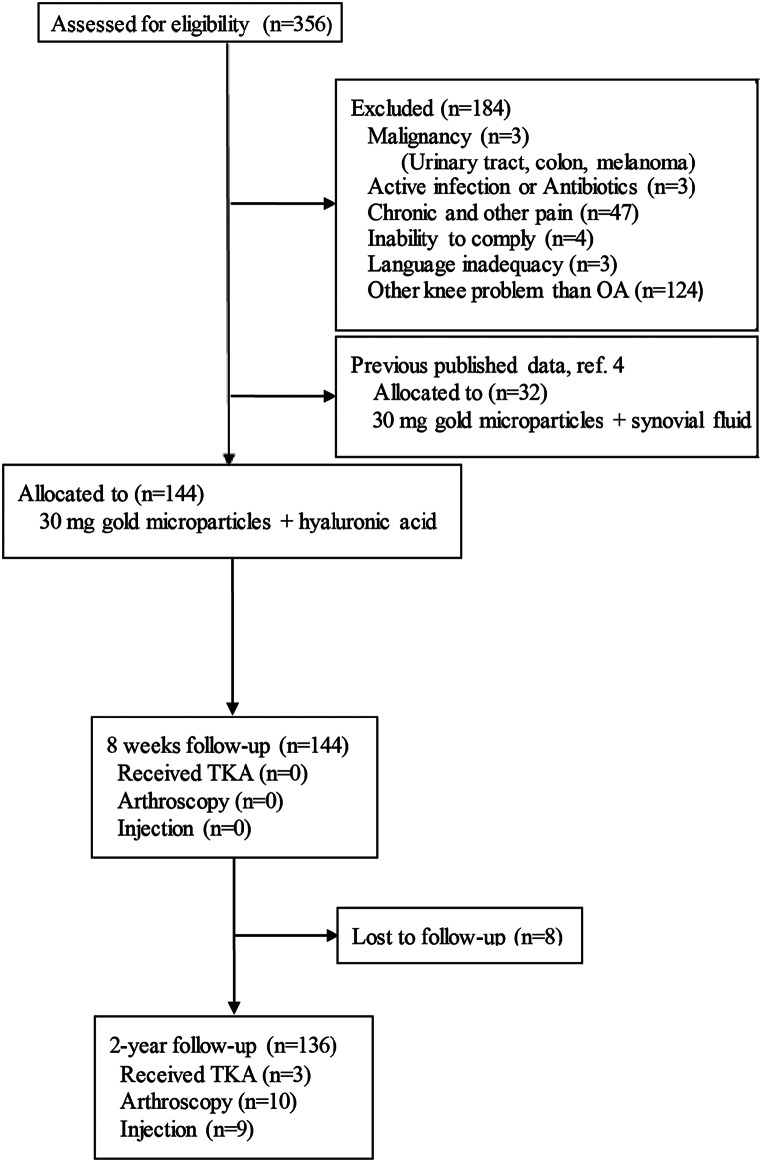
Flowchart of 136 knee osteoarthritic patients who received intra-articular injection of 20 mg gold micro-particles using hyaluronic acid

From January 2017 through March 2018, we enrolled 136 patients with radiographically confirmed KOA (Kellgren & Lawrence grade ≥ 1) [20], pain for more than three months, and maximal pain intensity VAS (Visual Analogue Scale, 0–10) ≥ 5 during the last week. We enrolled sligible patients at the specialized, public outpatient clinic at Aalborg University Hospital, Denmark. The exclusion criteria were (1) active adjuvant treatment for any malignancy, (2) active infection and antibiotic treatment, (3) active treatment with steroids or biological medication, (4) inability to comply with the protocol, and (5) inadequacy in written and spoken national language (Fig. 1).

### Study treatment

Pure gold particles, 20 mg sterile 99.99%, a total of 72.000 particles, 20–40 μm in diameter (BerlockMicroImplants (BMI), Berlock ApS) [21, 22] were injected intra-articular into the knee joint. Two ml HA (Suplasyn®, 20 mg/2 ml) was mixed with the sterile gold microparticles and injected into the patient’s knee.

### Primary outcome measures

Using the Global Rating of Change Scale [23], we asked the question, concerning your knee, how will you describe yourself compared to immediately before the injection of gold into your knee, and evaluated the answer on an 11-point scale from very much worse (-5) to complete recovered (5) with a score of zero indicating no changes.

### Secondary outcome measures

The PainDetect questionnaire (PDQ) [18] comprises three major components It is a gradation of pain, pain course pattern, and radiating pain. Seven questions evaluate the gradation of pain. The patient scored each question using a 0 to 5 score with 0 = never, 1 = hardly notice, 2 = slightly, 3 = moderately, 4 = strongly, and 5 = very strongly. There is one question evaluating pain course patterns. Patients select from one of four pictures indicating which best describes their course of pain. A unique score of 0, -1, or + 1 is associated with each picture). One question evaluates radiating pain with a yes (score of + 2) or no (score of 0) response option. PDQ is scored from 0 to 38, with total scores < 13 considered to represent nociceptive pain, 13–18 possible neuropathic pain, and > 18 representing neuropathic pain.

The Western Ontario and McMaster Universities Arthritis Index (WOMAC) sub-scores for pain, stiffness, and function [24], and contain 24 questions: 5 pain questions, 2 stiffness questions, and 17 physical function questions. Each question utilizes a 5-point scale, from 0 (none) to 4 (extreme).

### Explanatory variables for the outcome

The explanatory variables analyzed for the outcome are PDQ, age, sex, body mass index (BMI), and Kellgren & Lawrence grade.

### Statistical analysis

The Stata software, version 17.0 (StataCorp) was used when analyzing the clinical outcomes.

Ordinal logistic regression was used when analyzing the explanatory variables’ influence on the Global Rating of Change Scale, and the difference over time in WOMAC pain, stiffness, and function. The explanatory variables are PDQ, age, sex, body mass index (BMI), and Kellgren &Lawrence grade.

Wilcoxon test for trend was used for matched pairs test of before-after and Kruskal-Wallis test for between groups tests. The minimally clinically important difference for the Global Rating of Change Scale was defined as 2 [23], and for WOMAC pain, stiffness and function as, respectively, 4, 2 and 10 [25].

### Data availability

The datasets generated during the current study are available from the corresponding author upon reasonable request.

### Ethical approvals

The study followed the principles of the Declaration of Helsinki and was approved by the local ethics committee of the North Denmark Region by 27/07/2016 (N-20,160,045). The regional data protection agency approved the project by 06/07/2016 (2008-58-0028, ID 2016 − 116) and registered in ClinicalTrial.Gov by 04/01/2018 (NCT03389906).

We have followed the Consort guideline for reporting non-randomized pilot and feasibility studies [26]. The first author takes responsibility for the integrity and accuracy of the reported data and the credibility of the study to the protocol.

### Consent

All participants consented to participation in the research via written forms and verbally.

## Results

### Enrollment and follow-up

After assessment for inclusion, total of 136 patients were assessed for inclusion, and 136 were enrolled and underwent treatment with an intra-articular injection of 20 mg gold microparticles using hyaluronic acid as the carrier (Fig. 1). Table 1 presents the baseline characteristics. No patients presented with symptomatic accumulation or effusion needed aspiration, why hyaluronic acid was the carrier. All 136 patients completed the follow-up. During follow-up, three patients with the Kellgren &Lawrence grade IV [20] received total knee arthroplasty, ten patients had an arthroscopic procedure, and nine received additional intraarticular injection with gold micro-particles. All 136 patients were included in the analysis until censored due to additional treatment. The 22 patients who received additional treatment were included in the predictor analysis with a Global Rating of Change Score. The follow-up time was a mean of 25.1 months.

**Table 1:** Baseline characteristics of the 136 knee osteoarthritic patients receiving gold using HA. Values are median and range; and mean and 95% CI. Scores on the Kellgren–Lawrence scale range from 0 to 4, with a score of 1, 2, 3, or 4 indicating definite osteoarthritis and higher scores indicating more severe disease

Female/Male sex	70/66
Age–year	62 (28-91); 62 (39.1-85.3)
Body mass index	27.6 (18-42.9); 27.8 (17.6-38.1)
No MRI or clinical sign of effusion	22
Anti-inflammatory treatment	7
Multisite musculoskeletal pain	111
Both knee OA	46
Low back pain	36
Hip OA	21
Rotator cuff pain	8
Generalized OA	6
Fibromyalgia	4
Kellgren-Lawrence Score	
I	15
II	32
III	81
IV	18
Womac Scores	
Pain	9 (1-18); 9.3 (2.7-15.8)
Stiffness	5 (0-10); 4.4 (0.33-8.5)
Activity	30 (1-51); 27.5 (5.12-49.8)
Pain evaluation	
PDQ	10 (0-35); 10.5 (-2.7-23.6)
No < 13	92
No ≥ 13	44

### Primary outcome measures

On the 11-point Global Rating of Change Scale [23], at 2-year follow-up 94 (69.1%) patients reported a positive effect, and 39 (28.7%) reported no effect and 3 (2.2%) were worse (median 2 (-3; 5)). There was no difference in Global Rating af Change Scale from 8 weeks to 2-year follow-up.

### Secondary outcome measures

**Fig. 2:**
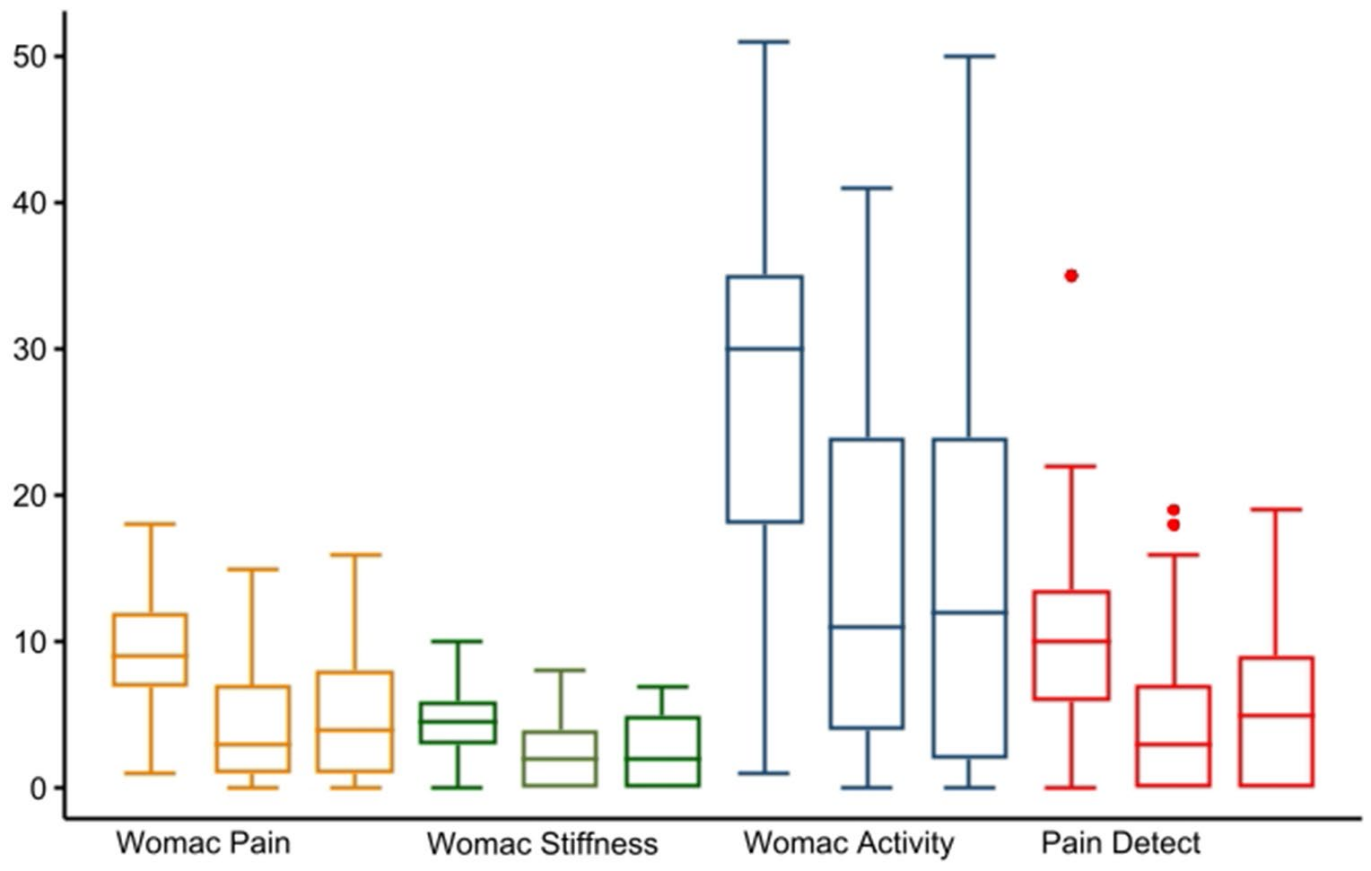
Change in womac pain, stiffness and activity, and pain detect questionnaire, before and 2 years after intra-articular injection of 20 mg gold micro-particles in 136 knee osteoarthritic patients (*P* < 0.01)

Compared to the baseline, the three WOMAC sub-scores [24] and PDQ [18] all improved at two years of follow-up (all *P* < 0.0001) (Fig. 2). WOMAC sub-scores at inclusion did not determine the effect (*P* > 0.5). There was no difference in WOMAC sub-scores from 8 weeks to 2-year follow-up.

### Explanatory variables for outcome

**Fig. 3:**
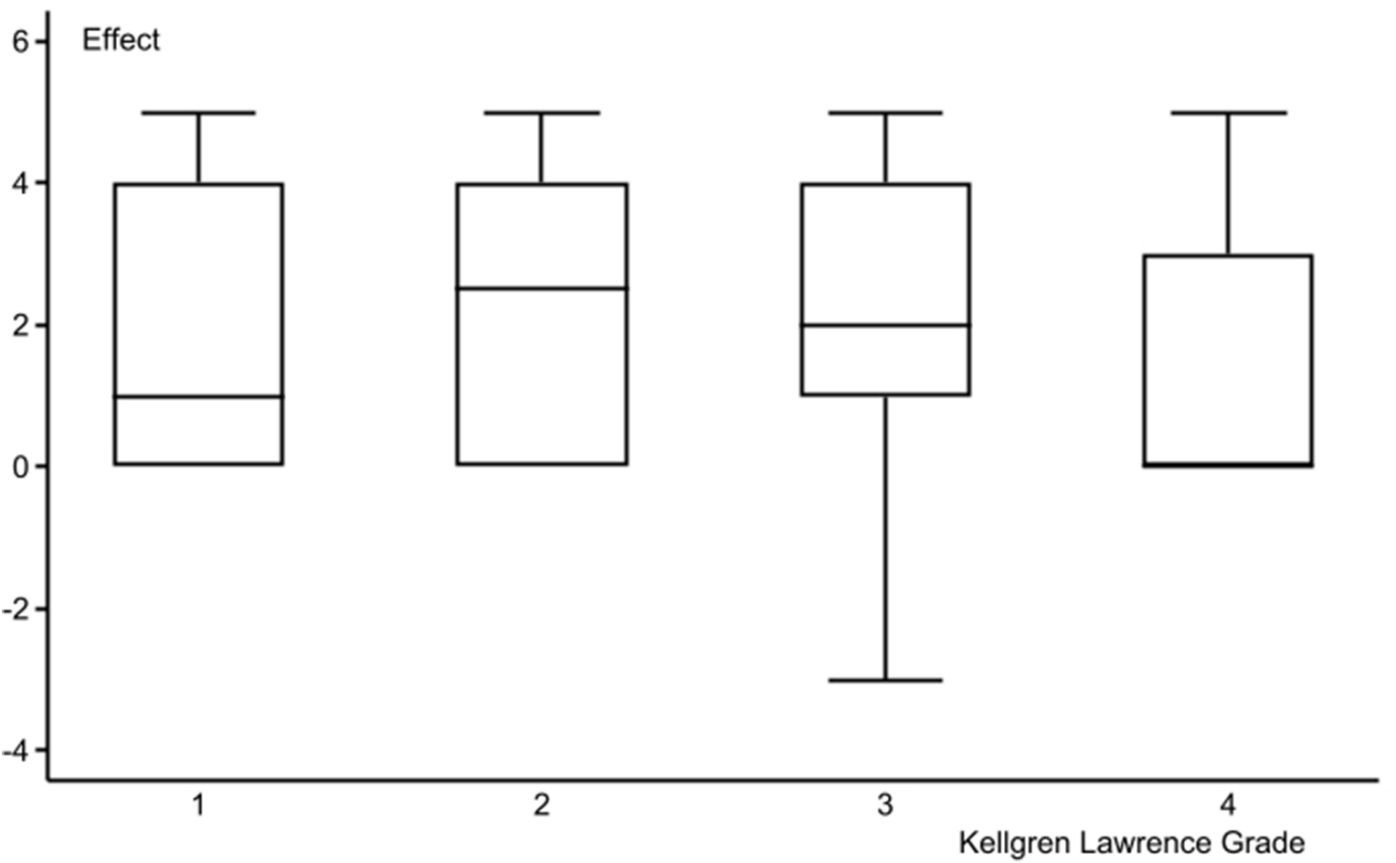
The association between Kellgren-Lawrence grade at inclusion and 2-year results after intra-articular injection of 20 mg gold micro-particles in 136 knee osteoarthritic patients (KL2, *P* = 0.04)

**Fig. 4:**
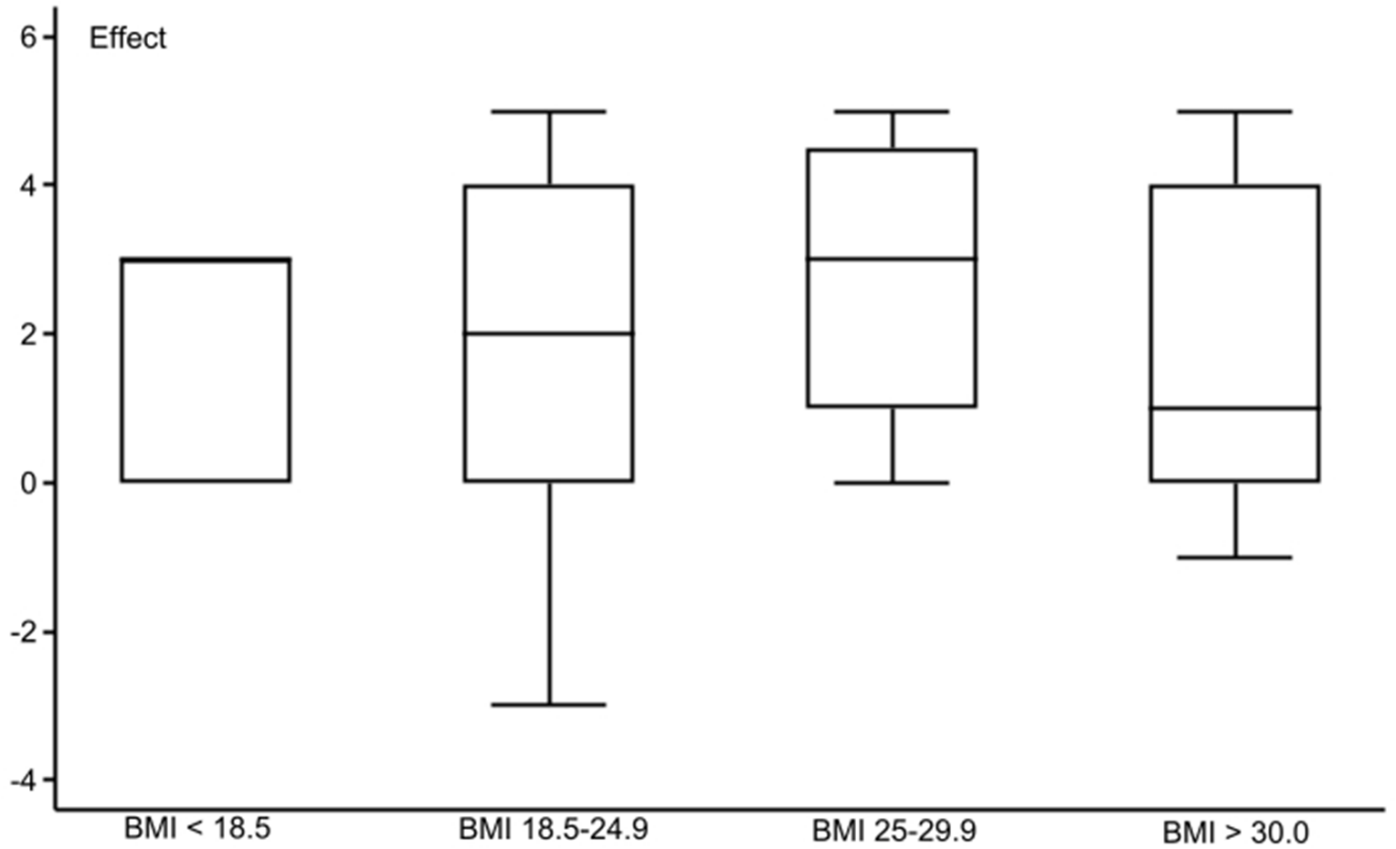
The association between BMI class at inclusion and 2-year results after intra-articular injection of 20 mg gold micro-particles in 136 knee osteoarthritic patients

**Fig. 5:**
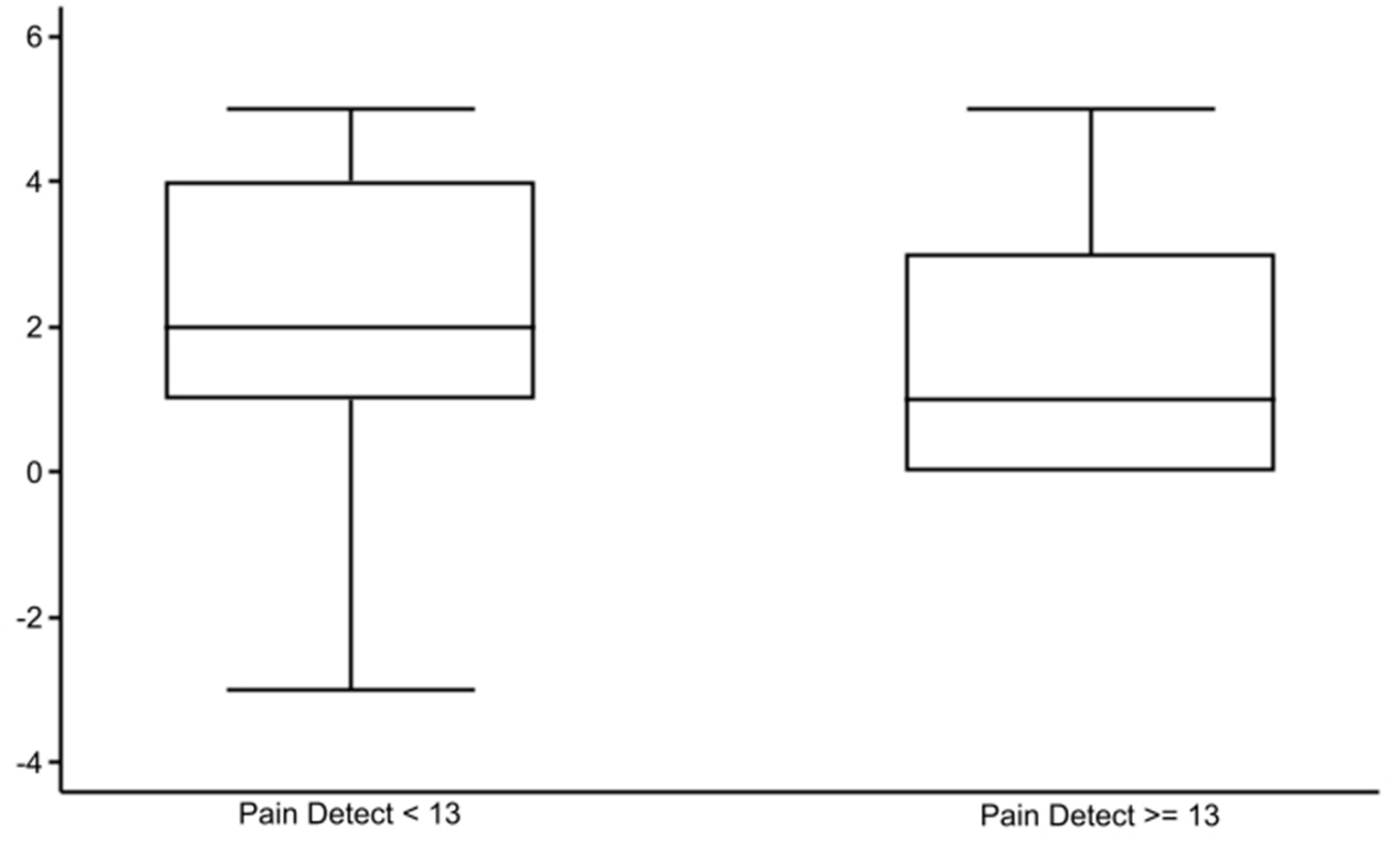
The association between Pain Detect Questionnaire at inclusion and 2-year results after intra-articular injection of 20 mg gold micro-particles in 136 knee osteoarthritic patients (*P* = 0.041)

The PDQ [18] scoring found that 1/3 of the patients had nociceptive pain with a score ≥ 13 (Table 1). In the ordinal logistic regression against the Global Rating of Change Score [23], PDQ ≥ 13, high BMI, and Kellgren &Lawrence grade 4 [20] at inclusion reduced the effect of intra-articular gold microparticles (Table 2). The odds ratio for a positive effect was lowest for Kellgren & Lawrence grade 4, followed by PDQ ≥ 13 and high BMI, with BMI being the most significant. Between-group analysis for these three explanatory variables confirmed the findings (Figs. 3 and 4, and 5). Figures 3 and 4 indicate a non-linear association of BMI and Kellgren & Lawrence grade at inclusion to the 2-year effect. Three was no difference in radiographic changes in patients with a PDQ < 13 compared to patients with a PDQ ≥ 13 (*P* = 0.28) or > 18 (*P* = 0.19) (Table 3). We found a negative odds ratio for improvement of stiffness in patients with a high BMI (*P* = 0.021).

**Table 2:** Logistic interval regression analysis of confounders at inclusion for outcome 2 years after intra-articular injection of 20 mg gold micro-particles in 136 knee osteoarthritic patients

Effect		Odds Ratio	P-value
Age		1.02 (0.98-1.06)	0.276
Female sex		0.44 (0.17-1.01)	0.078
BMI		0.9 (0.83-0.98)	0.022
Kellgren-Lawrence grade	II	1.42 (0.29-6.9)	0.668
	III	2.5 (0.61-10)	0.208
	IV	0.16 (0.03-0.82)	0.028
PDQ ≥ 13		0.35 (0.13-0.82)	0.028
Baseline odds		30.1 (0.69-1317)	0.025

**Table 3:** At inclusion distribution of the degree of neuropathic pain measured by Pain Detect Questionnaire (PDQ) about Kellgren-Lawrence degree of radiographic changes

Kellgren-Lawrence Grade	PDQ at inclusion		
	0-12	13-18	19-
1	11	3	1
2	11	8	3
3	57	20	4
4	13	5	0

## Discussion

This, non-placebo controlled, explorative study obtained individual patient data from included patients who received intra-articular gold microparticles for KOA using hyaluronic acid. Overall, the pattern of observed improvements was equal at the 2-year follow-up. We found a change in pain, stiffness, and activity, better than reported minimally clinically important differences [25].There was a significant improvement in pain and joint function in more than 2/3 of the patients. A neuropathic pain component (PDQ ≥ 13), obesity, and severe osteoarthritis reduce the effect of intra-articular gold microparticles for KOA.

Pain in osteoarthritis (OA) has few relations to specific radiological changes. There is generally a poor association between Kellgren &Lawrence and clinical pain intensity [13, 20] and individuals with osteoarthritic changes may not at all have pain [13, 20]. Gold ions may reduce pain, joint swelling, and inflammation [27–29], and the current study found that intra-articular gold microparticles improved pain and function, indicating that inflammation and many other factors [30], may be a cause of pain and disability. In addition, a molecular pathway that includes pro-inflammatory mediators may mediate pain [4, 31–33].

Chronic OA pain manifests with reduced pain threshold and general hyperalgesia due to sensitization processes [12, 14, 15]. We found PDQ [18] ≥ 13 in 33% of the patients, resembling sensitization and neuropathic pain. We found PDQ ≥ 13 associated with a poorer outcome of treatment. These results may indicate that patients with KOA demonstrate symptoms of neuropathic pain and sensitization. In a randomized study of intra-articular Botox for KOA-only patients with nociceptive pain, PDQ < 13, did benefit from the treatment [19]. A recent investigation of KOA patients with PDQ > 18 had fewer radiographic changes representing a specific phenotype of KOA [34], and the current study did not find a difference but a minimal tendency in radiographic changes concerning neuropathic pain, which indicates that more research is needed to identify the role for the PDQ in OA research.

The distribution of gold particles in the body varies depending on their size and route of administration [35]. Gold nanoparticles are used as contrast agents for X-ray and computed tomography scans, drug delivery and cancer therapy, and as carriers for therapeutic agents or as radiation enhancers. It is general knowledge that gold nanoparticles will enter an unknown multitude of cells, which might make long-term treatment problematic. Preclinical studies suggest that macrophages phagocytose gold nanoparticles intracellularly that oxidize in the lysosomes [35]. The macrophages remove the gold nanoparticles from the tissue as these cells move away over time.

Gold microparticles larger than 20 microns are too big for the macrophages to engulf or remove [35]. Therefore, the gold microparticles dissolve slowly by macrophages through dissolucytosis. The macrophages secrete cyanide compounds and dissolve the gold into cyanide-gold complexes such as Au (CN)^2−^ [21]. The gold microparticles stay put and continuously expose the matrix and the new cells to gold ions and influence the production of a new intercellular matrix. The fact that only cells close to the gold microparticles become loaded with gold implies that no gold ions are spread to gold-sensitive organs and exclude any toxic effects. Gold ions released from gold microparticles are a purely local process that slowly, andcontinuously produces local pharmaceutical levels of active gold ions into the intercellular space [4]. The use of gold microparticles, therefore, is considered a safe technique [35].

### Limitations

This exploratory study did not include a control or a placebo group and may overestimate the effect of intra-articular gold treatment. The patient’s expectations, the intention to treat the patient, and the puncture may influence the results. In addition, the relatively small number of patients may influence the results.

The use of WOMAC has limitations. As a composite score, it uses several questions integrated into a total score, not evaluating each question by itself. WOMAC and other composite scores may over- or underestimate the effect of treatment and intervention [36]. Studies indicate that WOMAC is more sensitive to changes in knee osteoarthritis symptoms and function than other scores, mainly due to a lower number of questions [37–39]. We used the WOMAC score as it has been validated in the context of knee osteoarthritis *and is considered a valid outcome measure by the Osteoarthritis Research Society International (OARSI) Standing Committee for Clinical Trials Response Criteria Initiative and the Outcome Measures in Rheumatology (OMERACT) committee* [40].

No patients were lost to follow-up. In addition, the intervention was carefully standardized and administered by the same physician (SR), the pain evaluation before and after eight weeks was done by the same research nurse (TJ), and the 2-year follow-up was performed by two medical students (ES and NKJ). We recorded no adverse effects related to the treatments. Any additional treatment events, TKA, arthroscopy, and injection were performed in 16% of cases during the two-year follow-up. In randomized studies on arthroscopy for degenerative changes, up to 36% in the control group received arthroscopy during follow-up [41]. In our study, 7% underwent knee arthroscopy. In randomized studies on TKA and non-surgical treatment of KOA, 14–32% underwent TKA replacement [42]. In our study, 2% underwent TKA. Regarding additional treatment after HA injection, such as reinjection, one study reports 50% reinjection within 12 weeks [43]. We found no placebo-controlled randomized studies on HA reporting additional arthroscopy or TKA. A review in 2016 [44] showed a lack of synthesis standardization leads to the opposite conclusion about the balance and benefits.

## Conclusions

A significant proportion of patients with knee osteoarthritis report a positive long-term effect of intra-articular injection of gold microparticles using hyaluronic acid. A neuropathic pain component (Pain Detect Questionnaire ≥ 13), obesity, and severe osteoarthritis hamper the effect. Compared to the results of our previous study [4], this study indicates that a part of the exclusion criteria used in our previous study may not be necessary for future studies. In addition, the current study found that 16% of patients needed additional treatment during follow-up. Future randomized, double-blinded studies need to identify the potential clinical use of intra-articular injection of gold microparticles for of knee osteoarthritis.

## Data Availability

The datasets used and analyzed during the current study are available from the corresponding author upon reasonable request.
